# Induction of breast cancer resistance protein by the camptothecin derivative DX-8951f is associated with minor reduction of antitumour activity

**DOI:** 10.1038/sj.bjc.6600508

**Published:** 2002-09-04

**Authors:** A H van Hattum, I J Hoogsteen, H M M Schlüper, M Maliepaard, G L Scheffer, R J Scheper, G Kohlhagen, Y Pommier, H M Pinedo, E Boven

**Affiliations:** Department of Medical Oncology, Vrije Universiteit Medical Center, De Boelelaan 1117, 1081 HV Amsterdam, The Netherlands; Division of Experimental Therapy, The Netherlands Cancer Institute, Plesmanlaan 121, 1066 CX Amsterdam, The Netherlands; Department of Pathology, Vrije Universiteit Medical Center, De Boelelaan 1117, 1081 HV Amsterdam, The Netherlands; Laboratory of Molecular Pharmacology, National Cancer Institute, NIH, Bethesda, Maryland, MD 20892, USA

**Keywords:** DX-8951f, BCRP, camptothecins, multidrug resistance, GF120918, ovarian cancer xenografts

## Abstract

DX-8951f (exatecan mesylate), a new water-soluble derivative of camptothecin, is currently being evaluated in phase II clinical trials. Resistance may be acquired when treating cancer patients with DX-8951f. Therefore, we selected a subline of the human ovarian cancer cell line A2780 for resistance against DX-8951f to investigate possible mechanisms of resistance. This DX-8951f-resistant subline, designated 2780DX8 (resistance factor=9.3), displayed a typical cross-resistance pattern including compounds, such as topotecan (resistance factor =34), SN-38 (resistance factor =47), mitoxantrone (resistance factor =59) and doxorubicin (resistance factor =2.9), which have previously been associated with the expression of breast cancer resistance protein. 2780DX8 cells did not show changes in the topoisomerase I gene, in topoisomerase I protein levels or catalytic activity. Overexpression of breast cancer resistance protein could be detected, both at the mRNA and protein level, while staining for Pgp, MRP1, or LRP was negative. GF120918, an inhibitor of breast cancer resistance protein, was able to reverse the DX-8951f-induced resistance in 2780DX8 cells. *In vivo* experiments in well-established 2780DX8 human tumour xenografts demonstrated that the growth inhibition induced by CPT-11 was more affected by breast cancer resistance protein expression than that of DX-8951f. These data indicate for the first time that DX-8951f is able to induce breast cancer resistance protein as a mechanism of resistance. Breast cancer resistance protein, however, results in only minor reduction of antitumour activity of DX-8951f which is an advantage over topotecan and CPT-11/SN-38.

*British Journal of Cancer* (2002) **87**, 665–672. doi:10.1038/sj.bjc.6600508 www.bjcancer.com

© 2002 Cancer Research UK

## 

DX-8951f, (1*S*,9*S*)-1-amino-9-ethyl-5-fluoro-2,3-dihydro-9-hydroxy-4-methyl-1*H*, 12*H*-benzo[de]pyrano[3′,4′:6,7]indolizino[1,2-*b*]quinoline-10-13(9*H*,15*H*)-dione methanesulphonate dihydrate (exatecan mesylate, Figure 1

**Figure 1:**
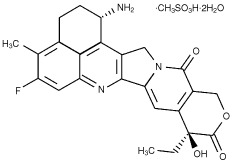
Structural formula of DX-8951f, (1*S*,9*S*)-1-amino-9-ethyl-5-fluoro-2,3-dihydro-9-hydroxy-4-methyl-1*H*, 12*H*-benzo[de]pyrano[3′,4′:6, 7]indolizino[1,2-*b*]quinoline-10-13(9*H*,15*H*)-dione methanesulphonate dihydrate.

), is a new water-soluble derivative of camptothecin. Its mechanism of action is through the inhibition of topoisomerase I activity. The potency of DX-8951f in various human malignant cell lines has been found to be six-fold and 28-fold greater than that of, respectively, SN-38 and topotecan (Mitsui *et al*, 1995). In addition, DX-8951f has shown efficacy in a variety of human tumour xenografts (Kumazawa *et al*, 1998). Phase II clinical trials on DX-8951f are currently in progress in Europe, the United States and Japan.

A major obstacle to successful anticancer therapy is the presence or development of drug resistance. Several mechanisms can confer resistance against topoisomerase I inhibitors, including reduction of topoisomerase I protein levels, decrease in the activity of the enzyme or the presence of drug transporters (Pommier *et al*, 1999). It has been suggested that DX-8951f is less affected by mechanisms of resistance described for other camptothecins. For example, DX-8951f has shown significant antitumour effects against cell lines and xenografts, in which CPT-11 was not effective (Takiguchi *et al*, 1997; Kumazawa *et al*, 1998). It may also overcome Pgp-mediated multidrug resistance, as it is highly effective against cell lines overexpressing the P170-glycoprotein transporter both *in vitro* and *in vivo* (Mitsui *et al*, 1995; Kumazawa *et al*, 1998).

A recently discovered drug transporter that appears to be relevant for camptothecin resistance is the breast cancer resistance protein (BCRP) (Doyle *et al*, 1998). BCRP is also known as placenta-specific ABC-transporter protein (ABCP) (Allikmets *et al*, 1998) and mitoxantrone-resistance (MXR) protein (Miyake *et al*, 1999). Overexpression of *BCRP* is characterised by a unique pattern of resistance. While resistance is observed against mitoxantrone, doxorubicin, and daunorubicin, cells remain sensitive to Vinca alkaloids, paclitaxel and cisplatin (Ross *et al*, 1999). *BCRP* is also involved in resistance against derivatives of camptothecin as cell lines overexpressing this transporter exhibit resistance against topotecan, SN-38 and 9-aminocamptothecin (Yang *et al*, 1995; Brangi *et al*, 1999).

We here describe for the first time that DX-8951f is able to induce *BCRP* and functional protein in the human ovarian cancer cell line A2780. The lowly resistant variant was designated 2780DX8. 2780DX8 cells were highly cross-resistant against mitoxantrone, topotecan and SN-38, but not against camptothecin or BNP1350. Resistance was not related to changes in topoisomerase I gene, protein levels or DNA-relaxation activity. GF120918, an efficient inhibitor of BCRP (de Bruin *et al*, 1999), was able to reverse the DX-8951f-induced resistance in 2780DX8 cells. Of interest, the growth inhibition induced by CPT-11 in 2780DX8 s.c. xenografts grown in nude mice was more affected by BCRP expression than that of DX-8951f.

## MATERIALS AND METHODS

### Drugs

DX-8951f (Daiichi Pharmaceutical Co., Tokyo, Japan), camptothecin (Aldrich, Milwaukee, WI, USA), SN-38 (Daiichi or Aventis Pharma, Vitry sur Seine, Cedex, France), BNP1350 (BioNumerik Pharmaceuticals, Inc., San Antonio, TX, USA), 10,11-methylenedioxy-20(*S*)-camptothecin (provided by Dr ME Wall, Research Triangle Institute, NC, USA) were prepared in DMSO (dimethylsulphoxide; Riedel-de Haën, Seelze, Germany). Topotecan (GlaxoSmithKline, Collegeville, UK) was diluted in water. CPT-11 was purchased as a solution from Aventis Pharma. Doxorubicin (Amersham Pharmacia, Roosendaal, The Netherlands) was dissolved in water. Solutions of cisplatin, paclitaxel and etoposide were obtained from Bristol-Myers Squibb (Woerden, The Netherlands), of 5-fluorouracil from Roche (Mijdrecht, The Netherlands) and of methotrexate from AHP Pharma (Hoofddorp, The Netherlands). Mitoxantrone was purchased from Sigma (St Louis, MO, USA) and was dissolved in PBS. GF120918, an inhibitor of Pgp and BCRP (Hyafil *et al*, 1993), was kindly provided by Dr Ken Brouwer (GlaxoSmithKline, Research Triangle Park, NC, USA) and dissolved in DMSO.

Drugs were further diluted in tissue culture medium when investigated for their antiproliferative effects *in vitro*. The final concentration of DMSO in culture did not exceed 1% (v v^−1^) which was non-toxic to the cells. When investigated *in vivo* CPT-11 was further diluted in NaCl 0.9% and DX-8951f was dissolved in water.

### Development of 2780DX8

The DX-8951f-resistant cell line, 2780DX8, was established from the human ovarian cancer cell line A2780 by continuously exposing cells to stepwise increasing concentrations of DX-8951f, starting at 0.1 nM to a final concentration of 8 nM after 7 months (approximately 30 passages). 2780DX8 was maintained in the presence of 8 nM DX-8951f. Both cell lines were grown in Dulbecco's modified Eagle's medium (Gibco-BRL, Breda, The Netherlands) supplemented with 10% heat-inactivated foetal calf serum (FCS; Gibco-BRL), 50 IU ml^−1^ penicillin and 50 μg ml^−1^ streptomycin (ICN, Zoetermeer, The Netherlands) in an incubator with a humidified atmosphere containing 5% CO_2_, at 37°C.

Doubling times were determined as follows. For each cell line a standard curve was obtained by plating a range of 500 to 100 000 cells per well in 12 replicate wells in a 96-well microtiter plate. After 2 h the MTT [(3-(4,5-dimethylthiazol-2-yl)-2,6-dimethyl-morpholino)-2,5-diphenyl-tetrazolium bromide; Sigma] assay was performed (see next section). Mean absorbances measured were plotted against the number of cells using regression analysis. Simultaneously, for all cell lines various cell numbers were plated in 12 replicate wells and grown for 24, 48, 72 and 96 h, after which the MTT assay was performed. Mean absorbances measured at each time-point were related to the cell number from the standard curves. Doubling times were calculated from the graph when the cells were in exponential growth phase. For each cell line at least nine separate doubling times were obtained and the mean doubling time was calculated.

### *In vitro* growth inhibition assay

Drug antiproliferative effects were determined with the MTT assay. Briefly, for A2780 and 2780DX8, respectively, 3000 and 5000 cells per well in 100 μl medium were plated in 96-well microtiter plates and grown for 24 h at 37°C. Cells were exposed continuously to serial dilutions of drug in a total volume of 200 μl for an additional 96 h. The medium was removed and 50 μl of the tetrazolium salt MTT in PBS (0.4 mg ml^−1^) were added. The plates were incubated for 2 h and formazan crystals were dissolved in 200 μl of DMSO per 0.5% FCS. The absorbance was measured at 540 nm using a Labsystems Multiscan Bichromatic plate reader (Labsystems, Helsinki, Finland). The results were expressed as the IC_50_, which is the concentration of drug inducing a 50% inhibition of cell growth of treated cells when compared to the growth of control cells. IC_50_ values were read from a graph expressing the concentration of drug against the percentage of relative growth. The ratio of IC_50_ resistant cells *vs* IC_50_ parent cells was presented as the resistance factor (RF). For reversal of BCRP-induced resistance against DX-8951f, SN-38, topotecan and mitoxantrone, GF120918 was administered at a non-toxic concentration of 2 μM just prior to adding the serial dilutions of the drugs. All concentrations were tested in four replicate wells and each experiment was performed at least three times.

### Northern blotting of *BCRP*

Total cellular RNA was isolated from exponentially growing cells using TRIzol (GibcoBRL). Ten μg of total RNA were fractionated on a 1% agarose-formaldehyde gel and subsequently transferred to nitrocellulose Hybond-N^+^ (Amersham Pharmacia). Blots were prehybridized for 1 h at 42°C in 5×SSC (1×SSC: 150 mM sodium chloride, 15 mM sodium citrate, pH 7.0), 5×Denhardt's solution, 0.2% SDS, 100 μg ml^−1^ salmon sperm DNA, and 50% deionized formamide. Subsequently, the blots were probed using 25 ng of the ^32^P-labeled *BCRP/MXR/ABCP* probe (Maliepaard *et al*, 1999) at 42°C overnight. After washing in 1×SSC/0.1% SDS for 20 min at room temperature and three times with 0.2×SSC/0.1% SDS for 10 min at 65°C, blots were analysed using a phosphor imaging system (Fujix Bas 2000; Fuji Photo Film Co. Ltd., Tokyo, Japan). Two separate RNA isolations of each cell line were examined.

### Gel electrophoresis and Western blotting

BCRP protein levels were determined using gel electrophoresis and Western blotting. Whole cell lysates were made by lysing harvested cells in 150 mM NaCl, 10 mM Tris-HCl (pH 7.6), 5 mM EDTA and 1% Triton X-100 supplemented with 1 mM PMSF, leupeptin (Sigma; 0.5 μg ml^−1^), and trypsin inhibitor (Sigma; 10 μg ml^−1^). Proteins were separated on 10% sodium dodecyl sulphate polyacrylamide gels using the Bio-Rad Mini Protean II cell system (Bio-Rad, Veenendaal, The Netherlands) and transferred to hybond polyvinylene difluoride membranes (Millipore, Bedford, MA, USA). The blots were blocked overnight with 5% Protifar (Nutricia, Zoetermeer, The Netherlands) in PBS at 4°C, washed and incubated with primary mouse monoclonal antibody (MAb) BXP-21 (Maliepaard *et al*, 2001a) for 2 h at room temperature. After washing, the blots were incubated with peroxidase-conjugated rabbit anti-mouse immunoglobulins (1 : 1000; Dako, Copenhagen, Denmark). Proteins were visualised using the ECL blotting detection reagents (Amersham Pharmacia). The expression levels were quantified using Scion Image for Windows (Scion Corporation) and six independent experiments were performed.

For determination of topoisomerase I protein levels nuclear protein extracts were prepared from cell lines as follows. 1×10^7^ Cells were lysed in nuclear buffer (150 mM NaCl, 1 mM KH_2_PO_4_, 5 mM MgCl_2_.6H_2_O, 1 mM EGTA, pH 6.4) supplemented with 0.3% Triton X-100, 0.8 mM phenylmethylsulphonyl fluoride (Merck, Amsterdam, The Netherlands) and 0.2 mM dithiotreitol (Sigma) and kept on ice for 10 min. Cell nuclei were harvested by centrifugation for 10 min at 1200 **g** (4°C) and washed with nuclear buffer. Nuclear proteins were extracted from the nuclei by incubation with nuclear buffer containing 0.4 M NaCl for 30 min on ice. After centrifugation at maximum speed the enzyme solution was diluted with an equal volume of 87% glycerol and stored at −70°C for a maximum of a week. Total protein concentrations of the nuclear extracts were determined according to Bradford. Proteins were separated on 7.5% sodium dodecyl sulphate polyacrylamide gels and transferred to hybond polyvinylene difluoride membranes. For detection of topoisomerase I a polyclonal human antibody to human topoisomerase I (1 : 5000; TopoGEN, Columbus, OH, USA) was used with Protein A/HRP (1 : 10 000; Amersham Pharmacia) as secondary reagent. The expression levels were quantified using Scion Image in three independent experiments.

### Immunocytochemistry

For detection of Pgp, MRP1, LRP and BCRP by immunocytochemistry the following MAbs were used. Pgp was determined using the murine MAb JSB-1 (IgG1), which recognizes an internal epitope of Pgp. For detection of MRP1 the mouse MAb MRPm6 (IgG1) was used. LRP expression was determined with the mouse MAb LRP-56 (IgG2b) and BCRP expression was assessed using the MAb BXP-34 (Scheffer *et al*, 2000). As positive controls the following cell lines were included: 2780AD (Pgp), GLC4/ADR (MRP1), 2R120 (LRP) and the MCF-7 MR cell line, overexpressing BCRP.

Staining for Pgp, MRP1, LRP and BCRP was performed on acetone-fixed (10 min) cytospin preparations. Slides were preincubated with 10% goat serum for 15 min and then incubated with primary antibodies diluted in phosphate-buffered saline (PBS) containing 1% (w v^−1^) BSA for 1 h. Antibody binding was detected using biotinylated goat anti-mouse IgG and streptavidin conjugated to horseradish peroxidase (Zymed, San Francisco, CA, USA). Bound peroxidase was visualised using the AEC Substrate Kit (Zymed). Slides were counterstained with haematoxylin (Merck) and mounted with Aquamount (BDH Laboratory Supplies, Poole, UK). Negative control slides were treated as above, substituting the primary antibody with a non-specific IgG control antibody or PBS. Staining intensities were categorised as negative (−), weakly positive (±), positive (+) or strongly positive (++). The staining intensities for the different proteins were determined in at least three independent experiments.

### Topoisomerase I catalytic activity

Topoisomerase I activity was determined by measuring the relaxation of supercoiled pHOT1 plasmid DNA (TopoGEN) according to Liu and Miller (1981). Serial dilutions of nuclear extract (0.16–40 μg ml^−1^) were added to a reaction mixture containing supercoiled pHOT1 plasmid and 125 mM Tris, 210 mM KCL, 25 mM MgCl_2_, 1.25 mM EDTA and 75 μg ml^−1^ BSA at pH 7.5 and incubated at 37°C for 30 min. The reaction was stopped by adding stop solution (30% (v v^−1^) glycerol 87%, 3% (w v^−1^) SDS, 0.3% (w v^−1^) bromophenol blue). Relaxed and supercoiled DNA were separated on a 1% agarose gel by electrophoresis and visualised by ethidium bromide staining. One unit of topoisomerase I activity was defined as the complete relaxation of 1 μg of supercoiled pHOT1 plasmid DNA per min at 37°C. DNA topoisomerase I activity was measured at least five times in each cell line.

### Detection of topoisomerase I gene mutations

For detection of possible mutations of the topoisomerase I gene RNA was extracted from the parental and drug-resistant cell line using a RNeasy Mini kit (Qiagen, Valenica, CA, USA) according to the manufacturer's protocol. RNA was reverse transcribed using the SuperScript preamplification system (Gibco-BRL). The three regions A, B, and D of the topoisomerase I cDNA, already known to bear mutations responsible for camptothecin resistance (Gupta *et al*, 1995), were amplified. PCR amplification was performed using the PCR Reagent system (Gibco-BRL) under the following conditions: preincubation was at 95°C for 2 min followed by 30 cycles at 95°C (1 min); 55°C (1 min); 72°C (1 min) and a final extension of 10 min at 72°C. Cloning of the PCR fragments was performed using the TOPO TA cloning system (Invitrogen, Carlsbad, CA, USA). Transformation of competent cells and DNA minipreps were performed according to standard procedures. Sequencing was performed using the BigDye primer cycle sequencing reaction kit (Applied Biosystems, Foster City, CA, USA). Analysis of the topoisomerase I sequences was performed using Sequencher software (Genes Code Corporation, Ann Arbor, MI, USA) and at least six separately obtained sequences were evaluated.

### *In vivo* antitumour efficacy

For the animal experiments, ethical approval was obtained from the ‘University Committee on Experimental Animals'. This Committee strictly adheres to the standards required by the UKCCCR for the welfare of animals in experimental neoplasia (Workman *et al*, 1998). The xenografts were established from cell lines grown in tissue-culture medium. Female nude mice (Hsd: athymic nude-*nu*; Harlan, Horst, The Netherlands) were inoculated subcutaneously with 1×10^7^ cells in both flanks. Upon growth, tumours were measured three times a week in three dimensions with vernier calipers. The volume was calculated by the equation length×width×thickness×0.5, and expressed in mm^3^. At the start of treatment (designated as day 0), groups of five to six tumour-bearing mice were formed to provide a mean tumour volume of approximately 250–300 mm^3^ in each group.

Doses of DX-8951f and CPT-11 for the daily ×5 schedule were administered according to the maximum tolerated dose (MTD) for non-tumour-bearing mice. This MTD was based on the occurrence of a mean reversible weight loss of approximately 10% of the initial weight within the first 2 weeks after the start of the treatment. CPT-11 was administered i.p. at a dose of 20 mg kg^−1^ daily×5, as established previously (Jansen *et al*, 1997). According to the MTD criteria the dose for the daily×5 schedule of DX-8951f was defined as 1.5 mg kg^−1^ i.p.

Three evaluation criteria were used in parallel to express the treatment efficacy: (1) mean tumour growth curves; (2) specific growth delay (SGD); and (3) relative tumour volume (RTV) on day 14. The SGD was calculated by comparing the two doubling times (T_2D_) of treated tumours and control tumours starting from day 0 according to the following formula:


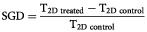


The RTV was calculated as the relative increase or decrease in mean tumour volume from the start of treatment (V_0_) until the value at a given time (V_t_) and expressed as the mean RTV (V_t_/V_0_).

### Statistics

Differences in *in vitro* drug sensitivities, *in vitro/in vivo* growth rate, and *in vivo* antitumour effects were evaluated using the two-tailed Students’ *t*-test for two samples with unequal variances. Data were normally distributed after log transformation and *P*-values <0.05 were considered to be significant.

## RESULTS

### Growth rate and cross-resistance pattern of 2780DX8

The doubling times (±s.e.m.) of A2780 and 2780DX8 cells were determined, showing that A2780 and 2780DX8 cells doubled in 11 h (±0.4) and 16 h (±1.0), respectively (*P*<0.001). The DX-8951f-resistant variant of A2780, 2780DX8, displayed a 9.3-fold resistance against DX-8951f when compared to the parental cell line A2780 (Table 1

**Table 1:**
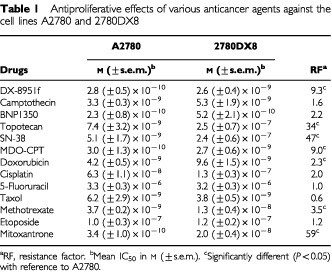
Antiproliferative effects of various anticancer agents against the cell lines A2780 and 2780DX8

). 2780DX8 cells were highly cross-resistant against the camptothecin derivatives topotecan (34-fold) and SN-38 (47-fold) and showed a moderate resistance against 10,11-methylenedioxy-20(*S*)-camptothecin (nine-fold). No cross-resistance against the parent compound camptothecin, or the camptothecin-analogue BNP1350 was observed. Evaluation of various other drugs, including doxorubicin, cisplatin, methotrexate, 5-fluoruracil, paclitaxel, etoposide and mitoxantrone, only showed high cross-resistance against mitoxantrone (59-fold) (Table 1). Minor cross-resistance was observed against doxorubicin (2.3-fold) and methotrexate (3.5-fold).

### Expression of Pgp, MRP1, LRP and BCRP

A2780 cells did not express any of the multidrug resistance proteins Pgp, MRP1, LRP, and BCRP, tested (Table 2

**Table 2:**
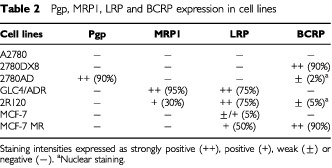
Pgp, MRP1, LRP and BCRP expression in cell lines

). 2780DX8 cells were negative for Pgp, MRP1 and LRP, but were strongly positive for BCRP. Approximately 90% of the 2780DX8 cells stained positive for BCRP. The cell lines used as a control for expression of Pgp (2780AD), MRP1 (GLC4/ADR), LRP (2R120) and BCRP (MCF-7 MR) indeed showed positive staining for these proteins.

**Figure 2:**
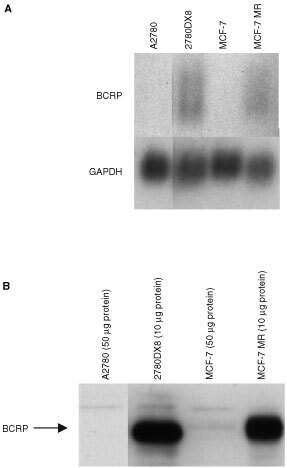
Northern blot of BCRP mRNA expression levels (**A**) and Western blot of BCRP protein levels (**B**) in A2780, 2780DX8, MCF-7 and MCF-7 MR. All lanes were cut from the same Northern or Western blot.

High expression of BCRP in 2780DX8 cells was confirmed on the mRNA and protein level using Northern blotting and Western blotting. Representative experiments are shown in Figure 2A and B

, respectively. No expression of BCRP could be detected in cell extracts from A2780. The control cell line MCF-7 showed very low levels of the protein on Western blot, but the probe used for Northern blotting was not sensitive enough to detect BCRP mRNA. MCF-7 MR cells, used as a positive control, showed high expression of BCRP.

### Reversal of BCRP-induced resistance

**Figure 3:**
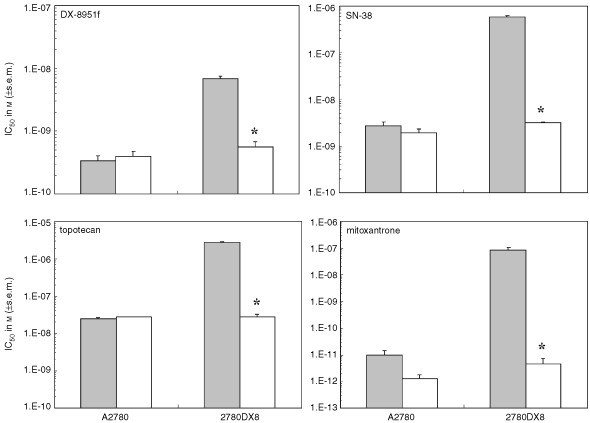
Reversal of BCRP-induced resistance against DX-8951f, SN-38, topotecan or mitoxantrone (grey bars) using 2 μM (white bars) GF120918. Antiproliferative effects were assessed using the MTT assay. The asterisks indicate a significant difference with reference to 2780DX8 without GF120918 (*P*<0.001).

In order to determine the contribution of BCRP expression to the resistance against DX-8951f in 2780DX8 cells, we used GF120918, a known antagonist of BCRP function, in a series of separate experiments. As can be seen in Figure 3

the resistance against DX-8951f could be reversed to the IC_50_ value of the parental cell line A2780 using 2 μM GF120918. GF120918 had no effect on the sensitivity of A2780 for DX-8951f. Of interest, the cross-resistance observed for SN-38, topotecan and mitoxantrone, which are all highly affected by BCRP, could also be fully reversed by GF120918.

### Topoisomerase I gene, protein and activity

**Figure 4:**
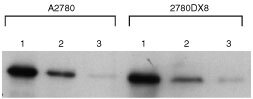
Western blot of topoisomerase I protein levels from A2780 and 2780DX8 cells; 2 μg (1), 1 μg (2) and 0.5 μg (3) nuclear protein was loaded for both cell lines.

The amount of topoisomerase I protein in nuclear extracts from 2780DX8 cells was similar to the amount of topoisomerase I in the parental cell line, A2780, as can be seen in a representative experiment depicted in Figure 4

. As compared to the relative intensity of A2780 defined as 1, the mean relative intensity (±s.e.m.) of 2780DX8 was 1.1 (±0.2). The catalytic activity of topoisomerase I measured in both A2780 and 2780DX8 cells by ATP-independent relaxation of supercoiled DNA demonstrated total relaxation of the plasmid in the presence of 2.5 μg ml^−1^ nuclear extract. The median activity of topoisomerase I in A2780 and 2780DX8 cells was similar and amounted to 1.3 u μg^−1^ nuclear protein (range 0.7–2.7 u μg^−1^).

The three regions of the topoisomerase I cDNA, already known to bear mutations responsible for camptothecin resistance (Gupta *et al*, 1995; Pommier *et al*, 1999) were analysed for changes in sequence when compared to the sequence found in A2780. No mutations were detected in the domains analysed (data not shown).

### *In vivo* activity of CPT-11 and DX-8951f

Determination of the growth rate (±s.e.m.) of untreated tumours revealed a slight difference in growth between A2780 and 2780DX8 xenografts as calculated starting from day 0 of the experiment. A2780-derived tumours doubled once in volume in 2.7 (±0.2) days and for 2780DX8-derived tumours the doubling time was 1.9 (±0.5) days (*P*<0.05). The growth rate of the tumours was also calculated from two-doubling times which was not significantly different between A2780 and 2780DX8 tumours. Immunohistochemistry was carried out using MAb BXP-34 on tissue sections of well-established tumours. Retention of BCRP was confirmed in 2780DX8, while no staining could be detected in A2780 xenografts (data not shown).

In the treatment experiments, drugs were given in equitoxic schedules on the basis of a similar degree of weight loss in non-tumour-bearing nude mice. When evaluating the weight loss induced by both compounds in tumour-bearing mice, we observed that DX-8951f was slightly more toxic than CPT-11. Recovery from weight loss, however, was complete on day 14 of the experiments. CPT-11 was hardly toxic in 2780DX8, which may be explained by weight gain from rapid tumour growth.

**Figure 5:**
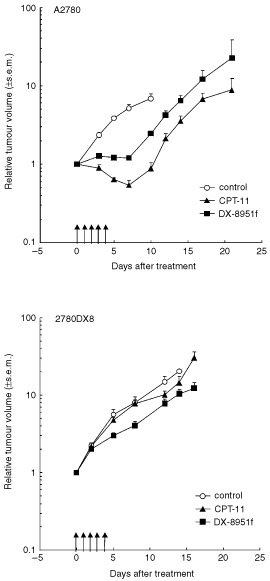
Growth curves of A2780 and 2780DX8 xenografts in nude mice representing the mean relative volume of control tumours, tumours treated with CPT-11 20 mg kg^−1^ i.p. daily×5 and tumours treated with DX-8951f 1.5 mg kg^−1^ i.p. daily×5. Arrows indicate the days of treatment.

Upon treatment it was clear that A2780 xenografts were more sensitive to CPT-11 than to DX-8951f (Figure 5

and Table 3

**Table 3:**
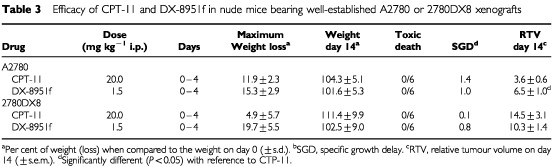
Efficacy of CPT-11 and DX-8951f in nude mice bearing well-established A2780 or 2780DX8 xenografts

). SGD values were 1.4 and 1.0, respectively. In contrast, the reverse was seen in 2780DX8 xenografts in which CPT-11 was not effective (SGD 0.1) and DX-8951f showed minor growth inhibition (SGD 0.8). The difference in efficacy was confirmed by the RTV values on day 14. The RTV upon treatment with DX-8951f was 1.8-fold higher in A2780 and 0.7-fold lower in 2780DX8 xenografts when compared to the respective values for CPT-11.

## DISCUSSION

In this report, we show for the first time that DX-8951f is able to induce the expression of BCRP as a mechanism of resistance, but the compound itself is hardly affected in antitumour activity by this transporter. The resistance against camptothecin analogues observed in BCRP-overexpressing 2780DX8 cells could not be related to changes in the topoisomerase I gene, in topoisomerase I protein levels or in its catalytic activity. In addition, we excluded the presence of other known multidrug resistance proteins. As the resistance against camptothecins as well as mitoxantrone could be completely reversed by GF120918, BCRP seems to be the only resistance mechanism present. Apart from our 2780DX8 cells, a few other cell lines have been described that show BCRP overexpression without change in topoisomerase I gene or protein. Maliepaard *et al* (1999) have selected a topotecan-resistant variant of the human ovarian cancer cell line IGROV-1, that overexpresses BCRP while topoisomerase I was not affected. A CPT-11- and a SN-38-selected subline of the lung cancer cell line PC-6, developed by Ishii *et al* (2000), were found to overexpress BCRP, without any changes in topoisomerase I. Although MRP1 levels were slightly higher in these cell lines as compared to the protein level in parental cells, no critical role was ascribed to MRP1 because inhibition of this transporter did not reverse resistance. It is of importance when BCRP is present to exclude other resistance mechanisms as the topotecan-selected human breast cancer cell line MCF-7, described by Yang *et al* (2000), showed concurrent reduction of the topoisomerase I protein content.

The difference in the degree of resistance against the various camptothecins observed in 2780DX8 cells might give information on the substrate affinity for BCRP. 2780DX8 cells were highly resistant against SN-38 and topotecan, 47- and 34-fold respectively. A lower level of resistance was observed against DX-8951f and 10,11-methylenedioxy-20(*S*)-camptothecin, respectively 9.0- and 9.3-fold, while the activity of both camptothecin and BNP1350 was not changed. A similar resistance profile has been described for the other BCRP-overexpressing sublines selected for resistance against camptothecin derivatives, in which topoisomerase I was not affected (Ishii *et al*, 2000; Maliepaard *et al*, 2001c). We can confirm that lipophilicity of the derivatives does not seem to play a direct role in the affinity for BCRP. Although the patterns of cross-resistance against camptothecins in BCRP-overexpressing sublines show similarities, absolute substrate affinity values should be calculated by measuring cellular transport kinetics of the various compounds.

Brangi *et al* (1999) have suggested that compounds, which are glucuronidated in the course of their metabolism, are most likely affected in activity in cancer cells with the BCRP phenotype. Glucuronidation is known for SN-38 (Rivory and Robert, 1995) as well as topotecan (Rosing *et al*, 1998), and both have shown to be substrates for BCRP. There are no reasons, however, to believe that DX-8951f will be glucuronidated. This compound has no hydroxyl group, which is known to be easily glucuronidated (Tephly and Burchell, 1990). Furthermore, no glucuronidated metabolites of DX-8951f have been found until now (Dr A Tohgo, Daiichi: personal communication). Consistent with Brangi *et al* (1999), we conclude that glucuronidation is not a requirement for BCRP substrates and that other molecular properties should also be taken into account to clarify the affinity of compounds for BCRP.

Of interest, minor cross-resistance against the antifolate methotrexate was observed in 2780DX8. Volk *et al* (2000) have recently investigated the role of BCRP in the resistance against methotrexate in the BCRP-overexpressing breast cancer cell line MCF7/MX, which exhibits high cross-resistance against this drug. They concluded that BCRP does not seem to be involved in methotrexate resistance, as BCRP-transfected MCF7 cells did not display cross-resistance, suggesting the presence of a novel MTX-specific efflux pump.

Presently, not much is known about the physiological role of BCRP. Normal tissue distribution of BCRP mRNA has demonstrated the highest expression in placental tissue and considerably lower expression levels are present in brain, prostate, small intestine, testis, ovary, colon and liver (Doyle *et al*, 1998). In one study low expression has been observed in kidney and heart as well (Allikmets *et al*, 1998). Immunohistochemistry has shown that prominent staining of BCRP is present in placental syncytiotrophoblasts, in the epithelium of the small intestine and colon, in the liver canalicular membrane, in ducts and lobules of the breast and in venous and capillary, but not arterial endothelium (Maliepaard *et al*, 2001a). This distribution in normal tissues resembles the distribution of Pgp (Ambudkar *et al*, 1999) and may point towards a physiological role comparable to Pgp, such as a protective function in normal tissues and fetus against toxic agents, regulation of uptake of orally administered substrates, and excretion processes in the liver.

In addition to the expression of BCRP in normal human tissues, there is some information available on its expression in human tumour samples. Ross *et al* (2000) have shown that BCRP mRNA could be detected in blast cells obtained from acute myeloid leukemia patients, although expression levels varied more than 1000-fold among samples tested. Scheffer *et al* (2000) have examined the protein expression of BCRP in a panel of 57 tumour samples derived from a variety of primary and pretreated tumours and found only one case of small-intestine cancer to be weakly positive. In contrast, Izquierdo *et al* (2001) have analysed 120 non-pretreated tumour samples of 20 different tumour types and detected BCRP positivity in over 40% of the samples (positive cases per tumour type 3–15). Furthermore, Maliepaard *et al* (2001b) have analysed 10 samples each of colon carcinoma, ovarian carcinoma, non-small cell lung cancer (NSCLC) and small cell carcinomas of different origin. In general, low staining was observed and expression was detected in three out of 10 colon carcinomas and in six out of 10 NSCLC. It thus appears that BCRP can be present in human tumours. Further data have to be awaited whether BCRP can be induced as a result from treatment.

Our data suggest that the presence of BCRP in tumours could have important consequences for the activity of several camptothecin derivatives and future analogues. Encouraging is the fact that, unlike topotecan and CPT-11/SN-38, DX-8951f is only slightly affected in antitumour activity, even though the expression of BCRP was generated by DX-8951f. In addition, the natural occurrence of BCRP in the small intestine will limit the oral bioavailability of camptothecins that are highly affected by this transporter. For the development of new (oral) derivatives of camptothecin aiming at an improved therapeutic index, it is of advantage to design molecules not extruded by BCRP.
